# Seropositividad de *Chlamydia psittaci* en trabajadores expuestos a aves y revisión de la literatura: evidencia de circulación en Antioquia

**DOI:** 10.7705/biomedica.6832

**Published:** 2023-09-30

**Authors:** Ana Claudia Ossa-Giraldo, Xiomara Úsuga-Perilla, Jhon Sebastián Correa, Juan A. Segura

**Affiliations:** 1 Grupo Infettare, Facultad de Medicina, Universidad Cooperativa de Colombia, Medellín, Colombia Universidad Cooperativa de Colombia Universidad Cooperativa de Colombia Medellín Colombia; 2 Grupo Biociencias, Facultad de Ciencias de la Salud, Institución Universitaria Colegio Mayor de Antioquia, Medellín, Colombia Institución Universitaria Colegio Mayor de Antioquia Medellín Colombia

**Keywords:** psitacosis, Chlamydophila psittaci, salud única, estudios seroepidemiológicos, inmunoglobulina G, inmunoglobulina M, exposición profesional, aves, Psittacosis, Chlamydophila psittaci, One Health, seroepidemiologic studies, immunoglobulin G, immunoglobulin M, occupational exposure, birds

## Abstract

**Introducción.:**

La psitacosis es una enfermedad zoonótica causada por *Chlamydia psittaci*. Esta bacteria es catalogada como un agente con potencial bioterrorista y ha causado múltiples brotes en trabajadores con exposición laboral a aves en diferentes lugares del mundo. En Colombia, no se hace seguimiento epidemiológico de la infección y existe una gran brecha en el conocimiento.

**Objetivos.:**

Determinar la frecuencia de anticuerpos contra *C. psittaci* en trabajadores con exposición laboral a aves y sus factores asociados. Además, revisar la literatura en relación con los estudios sobre el tema realizados en Colombia.

**Materiales y métodos.:**

Se llevó a cabo un estudio descriptivo, transversal, con intención analítica, en trabajadores en contacto con aves y se revisó la literatura científica relacionada en Colombia. Se detectaron anticuerpos IgM e IgG contra *C. psittaci* en suero por microinmunofluorescencia. La descripción de las características sociodemográficas y de exposición se hizo con frecuencias y medidas de resumen. Se exploraron factores asociados por análisis bivariados y multivariados. La revisión de la literatura científica y gris se hizo con búsqueda estructurada.

**Resultados.:**

Se analizaron 54 trabajadores en contacto con aves y se encontró una prevalencia de anticuerpos del 31,5 %. El ejercer funciones de sacrificio y faenado de las aves sin ser médico veterinario fue un factor de riesgo para la presencia de anticuerpos. Solo se encontraron cuatro estudios previos sobre *C. psittaci* hechos en Colombia.

**Conclusiones.:**

Este estudio constituye la primera evidencia de la circulación de *C. psittaci* en trabajadores en contacto con aves en Antioquia y el segundo reporte en el país. Estos hallazgos aportan desde la salud pública a la estrategia *One Health*.

## Abstract (en)

**Introduction.:**

Psittacosis is a zoonotic disease caused by *Chlamydia psittaci*, a bacterium classified as an agent with bioterrorist potential. It has caused multiple outbreaks in exposed poultry workers around the world. Colombia has no epidemiological follow-up of the infection and a big knowledge gap.

**Objectives.:**

To determine the antibodies’ frequency against *C. psittaci* in workers with occupational exposure to birds and to review the literature on studies conducted in Colombia.

**Materials and methods.:**

We conducted a cross-sectional descriptive study with analytical intent on workers in contact with birds and reviewed the related literature in Colombia. IgM and IgG serum antibodies against *C. psittaci* were detected by microimmunofluorescence. The sociodemographic and exposure characteristics were expressed as frequencies and summary measures. Associated factors were explored by bivariate and multivariate analysis. The scientific and gray literature review was done with a structured search.

**Results.:**

We analyzed 54 workers in contact with birds. Antibody prevalence was 31.5%. Slaughtering and evisceration by non-veterinarians was a risk factor for antibody presence. There are only four previous studies on *C. psittaci* in Colombia.

**Conclusions.:**

Here, we present the first evidence of *C. psittaci* circulation among workers exposed to birds in Antioquia and the second report in the country. These findings contribute to the “One Health” public health strategy.

*Chlamydia psittaci* es una bacteria gramnegativa, intracelular obligada, ^1^, que causa una infección generalizada con sintomatología respiratoria, denominada ornitosis o clamidiosis aviar ^1^^-^^3^. Se ha demostrado que esta bacteria puede infectar a más de 465 especies de aves, incluyendo psitácidas, palomas, pavos, patos y pollos, entre otras ^3^. Sin embargo, también se han reportado otras aves silvestres y mamíferos como reservorios de la bacteria ^4^^,^^5^. Factores como el hacinamiento, la manipulación, los ectoparásitos, la puesta de huevos, las deficiencias alimenticias y la exposición a ambientes contaminados con la bacteria favorecen la infección entre los animales ^1^^,^^4^.

*Chlamydia psittaci* se encuentra catalogada como un agente bioterrorista por su potencial zoonótico, gran capacidad de transmisión, dispersión y virulencia ^6^. En el mundo se han reportado grandes tasas de morbilidad- mortalidad humana ^7^. La transmisión zoonótica de *C. psittaci* se produce por contacto con secreciones respiratorias, tejidos o excrementos provenientes de las aves infectadas; en los humanos, causa la enfermedad denominada psitacosis ^7^^,^^8^. Esta infección afecta principalmente el sistema respiratorio y puede manifestarse por un cuadro clínico leve de tipo resfriado, complicarse como una neumonía atípica o diseminarse a otros órganos, causando una enfermedad sistémica que en algunos casos conduce a la muerte ^9^.

Entre los años 1929 y 1930, se reportó una epidemia de psitacosis que causó cerca de 800 casos y un centenar de muertes en humanos ^10^ y, hasta la fecha, se han reportado múltiples casos en trabajadores o personas que tienen contacto con aves infectadas, o manipulan sus fómites o restos ^11^. En el 2017, se reportó en Argentina el diagnóstico de psitacosis en ocho humanos y la presencia de *C. psittaci* en cuatro aves relacionadas con los mismos ^12^. En los últimos años, en diversos estudios se ha evidenciado la presencia de la bacteria o de anticuerpos contra ésta en aves en cautiverio en Venezuela ^13^, Ecuador ^14^, Chile ^15^ y Brasil ^16^, lo que demuestra su circulación actual en la región.

Los *Centers for Disease Control and Prevention* (CDC) han clasificado a *C. psittaci* en la categoría B de agentes con potencial de amenaza en la guerra biológica y, a la psitacosis, como de notificación obligatoria para los Estados Unidos ^17^^-^^19^. Además, recomiendan la implementación de estas categorizaciones en todo el mundo para optimizar los programas de prevención y control de los agentes infecciosos zoonóticos, mediante el enfoque multisectorial *One Health*, para la respuesta a brotes y el control de enfermedades que afectan a humanos, animales y el medio ambiente ^19^^,^^20^.

La detección de *C. psittaci* ha mejorado en la actualidad con el uso de pruebas moleculares rápidas y se espera que terminen por imponerse. Sin embargo, no están disponibles en todos los laboratorios y, por lo tanto, las pruebas serológicas, como la microinmunofluorescencia y la prueba ELISA (*Enzyme-Linked Immunosorbent Assay*) siguen siendo las de primera elección en el mundo ^7^^,^^21^^,^^22^.

A pesar de la importancia del seguimiento de la infección en humanos y la circulación de *C. psittaci*, desde la salud pública y la individual ^19^, en Colombia la psitacosis no es de notificación obligatoria ^23^ y, más aún, no es una enfermedad de diagnóstico rutinario en el humano. No obstante, la clamidiosis aviar sí es de notificación obligatoria en el país ^24^. En el estudio publicado en el 2011 por Monsalve *et al*., se indicó una seroprevalencia del 78 % de *C. psittaci* en trabajadores colombianos en contacto con aves ^25^.

Teniendo en cuenta que ese es el primer reporte conocido de *C. psittaci* en el país y la escasa evidencia en la literatura colombiana, es obvio el vacío en el conocimiento sobre la bacteria, la infección que produce y la exposición a la misma de los trabajadores en contacto con aves.

Esta investigación tuvo como objetivos: (i) determinar la frecuencia de anticuerpos contra *C. psittaci* en individuos expuestos laboralmente al contacto con aves y explorar la asociación de la presencia de anticuerpos con las características sociodemográficas y de exposición laboral; y (ii) realizar una revisión de la literatura indexada y gris para tener un panorama de la circulación de *C. psittaci* en Colombia.

## Materiales y métodos

### Diseño y población de estudio

Se llevó a cabo un estudio descriptivo transversal con intención analítica, con 54 trabajadores de centros de recuperación o atención de fauna silvestre y animales de compañía, policía ambiental, entidades de vigilancia y control avícola, y centros de comercialización de aves de Medellín (Colombia).

El muestreo fue no probabilístico a conveniencia. Se incluyeron individuos que tuvieron contacto con aves durante sus funciones laborales. Todos los sujetos fueron informados del estudio y sus riesgos, aceptaron participar voluntariamente y firmaron un consentimiento informado antes de su inclusión en el estudio.

### Plan de recolección de la información

*Información sociodemográfica y clínica:* a los participantes se les hizo una encuesta asistida con preguntas dicotómicas y politómicas, para determinar sus factores sociodemográficos, su exposición laboral y doméstica a aves, e indagar sobre sintomatología clínica durante las dos semanas anteriores a la inclusión en el estudio, tanto de los participantes como de las aves con las que tuvieron contacto.

*Pruebas inmunológicas:* a cada individuo se le tomó una muestra de 15 ml de sangre total sin anticoagulante para separar el suero y almacenarlo a -20 °C hasta su procesamiento. Los anticuerpos IgM e IgG contra *C. psittaci* se detectaron mediante la técnica de microinmunofluorescencia (*Chlamydia pneumoniae* IFA IgM e IgG, Vircell®, España), siguiendo las indicaciones del fabricante.

Esta prueba permite la detección específica de anticuerpos contra tres especies de *Chlamydia: C. pneumoniae*, *C. trachomatis* y *C. psittaci*. Se basa en el uso de elementos específicos de cada especie y libres de lipopolisacáridos, en pozos independientes para evitar las reacciones cruzadas y los falsos positivos ^26^. Los sueros que evidenciaron anticuerpos se titularon hasta obtener la mayor dilución de reacción. Se detectaron los anticuerpos IgM e IgG contra las tres especies. Sin embargo, para efectos del presente estudio, solo se reportan los resultados de *C. psittaci*.

*Búsqueda bibliográfica:* se hizo una búsqueda estructurada de la literatura científica y gris para identificar los estudios sobre psitacosis o *C. psittaci* en Colombia. La búsqueda se hizo en las bases de datos PubMed, Science Direct y Scielo. Para la búsqueda de literatura gris, se usó el motor Google. Las estrategias de búsqueda fueron (i) *Chlamydia psittaci*; (ii) *Chlamydia psittaci* AND Colombia; y (iii) Psittacosis AND Colombia.

Las búsquedas se llevaron a cabo en inglés y en español, sin filtro de años. De los resultados obtenidos, se seleccionaron únicamente los estudios hechos en Colombia, tanto en humanos como en aves.

### Análisis estadístico

La información se sistematizó y analizó en el software estadístico IBM SPSS™ Statistics, versión 25. El análisis descriptivo se hizo con frecuencias y medidas de resumen. Para el análisis bivariado, se usó la prueba de ji al cuadrado, la prueba exacta de Fisher y la U de Mann-Whitney. Se utilizó regresión logística binaria para el análisis multivariado. El valor de p<0,05 se consideró significativo.

### Aspectos éticos

Esta investigación se realizó siguiendo las directrices de la Resolución 8430 de 1993 y la Declaración de Helsinki. El estudio fue aprobado por el Subcomité de Bioética en Investigación de la Universidad Cooperativa de Colombia, sede Medellín, informe de aprobación 0800-0003.

## Resultados

Se incluyeron 54 trabajadores expuestos a aves durante sus funciones laborales. El 79,6 % (n=43) eran hombres y el 20,4 % (n=11) mujeres, con una mediana de edad de 33 años (rango intercuartílico = 29,75-47,50). El 35,2 % (n=19) eran profesionales o tenían título de posgrado, y el 24,1 % (n=13), técnicos o tecnólogos, el 38,9 % (n=21) había terminado su educación básica o media (primaria y bachillerato) y un individuo reportó no tener estudios.

Para tener un panorama de la exposición a aves durante el trabajo, se indagó a los participantes por los cargos que desempeñaban, sus funciones y la frecuencia del contacto con las aves. Asimismo, si habían presentado sintomatología respiratoria indicativa de psitacosis en las dos últimas semanas previas a la inclusión en el estudio. El 40,7 % (n=22) era comerciante de aves; el 25,9 % (n=14), médicos veterinarios, y el 18,5 % (n=10), policías ambientales y ecológicos. El porcentaje restante desempeñaba otras ocupaciones en las que ocasionalmente manipulaban aves, como labores administrativas, educación ambiental, coordinación de equipos de trabajo y labores exclusivas de transporte (cuadro 1).

**Cuadro 1:** Funciones laborales, frecuencia del contacto con las aves y síntomas en aves y trabajadores

Ocupación^a^	n	%
Médico veterinario	14	25,9
Zootecnista	4	7,4
Policía ambiental y ecológico	10	18,5
Trabajador en comercio de aves	22	40,7
Otras ocupaciones^b^	6	11,1
Funciones laborales^c^	n	%
Atención médica o diagnóstica de aves	14	25,9
Limpieza de los desechos de aves	31	57,4
Transporte de aves	25	46,3
Liberación de aves	9	16,7
Comercialización de aves	21	38,9
Vigilancia y cuidado de aves	31	57,4
Sacrificio y faenado de aves	19	35,2
Frecuencia del contacto con las aves durante las funciones laborales	n	%
Diario	29	53,7
4 a 6 días a la semana	4	7,4
2 a 3 días a la semana	11	20,4
2 a 3 veces al mes	6	11,1
1 vez al mes	3	5,5
Esporádicamente	1	1,9
Síntomas del trabajador^d^	n	%
Fiebre	6	11,1
Tos	19	35,2
Dolor en el pecho	5	9,3
Dificultad para respirar	8	14,8
Pérdida repentina de peso	1	1,9
Expectoración	10	18,5
Sudoración	6	11,1
Escalofríos	5	9,3
Reporte de síntomas evidenciados en las aves^e^	n	%
Disminución de la actividad	21	38,9
Plumas erizadas	27	50,0
Dificultad para respirar	13	24,1
Secreciones en ojos y fosas nasales	10	18,5
Diarrea con excremento amarillo o verde	12	22,2

^a^ Hubo dos personas que eran tanto médicos veterinarios como zootecnistas

^b^ Personas que tienen cargos administrativos, por ejemplo: educador ambiental y coordinador, entre otros.

^c^ Un individuo puede realizar una o más funciones.

^d^ Síntomas que presentó el trabajador durante las dos últimas semanas previas al estudio. Un trabajador puede presentar uno o más síntomas.

^e^ Síntomas que el trabajador reportó observar en las aves con las que estuvo en contacto laboral durante las dos últimas semanas previas al estudio. Un trabajador puede reportar uno o más síntomas observados en las aves.

Entre las funciones cumplidas durante el trabajo, las reportadas con mayor frecuencia por los participantes fueron, en igual medida, la vigilancia y cuidado de las aves y la limpieza de los desechos 57,4 % (n=31), seguidas del transporte 46,3 % (n=25). El 35,2 % (n=19) reportó cumplir labores de sacrificio y faenado de las aves. Respecto a la frecuencia del contacto con las aves, el 81,5 % (n=44) de los trabajadores reportó un contacto diario o de varias veces a la semana (cuadro 1). El 61,1 % (n=33) indicó al menos un síntoma respiratorio; los de mayor frecuencia fueron: tos (35,2 %; n=19), expectoración (18,5 %; n=10) y dificultad para respirar (14,8 %; n=8) (cuadro 1).

Se investigó sobre el tipo de aves a las que estaban expuestos los trabajadores en su entorno laboral, si tenían aves de compañía y si alguna de estas aves había presentado síntomas sugestivos de ornitosis en las dos semanas previas al estudio. Se reportaron contactos con 19 tipos de aves diferentes en el entorno laboral: periquitos (70,4 %; n=38), gallinas (68,5 %; n=37), cacatúas (59,3 %; n=32), palomas (51,8 %; n=28), patos (48,1 %; n=26), loros (42,6 %; n=23), guacamayas (38,9 %; n=21), pavos (37,0 %; n=20) y gaviotas (18,5 %; n=10). Se reportaron frecuencias menores del 12 % para el contacto con alondras, canarios, aves rapaces, bengalíes, cotorras, nodrizas, monjes, Fischer, sinsontes y turpiales. El 64,8 % (n=35) de los trabajadores reportó haber observado, al menos, un síntoma en las aves de su entorno laboral. Los más frecuentes fueron plumas erizadas (50,0 %; n=27), disminución de la actividad (38,9 %; n=21) y dificultad para respirar (24,1 %; n=13) (cuadro 1).

El 29,6 % (n=16) de los trabajadores reportó tener aves de compañía y encargarse de la alimentación, el cuidado y la limpieza de sus desechos. De estos, el 62,5 % (n=10) indicó tener periquitos, el 25,0 % (n=4), cacatúas, el 18,8 % (n=3), loros, el 12,5 % (n=2), canarios o guacamayas, y el 6,25 % (n=1), pollos o palomas. Solo 7,8 % (n=5) de los trabajadores reportó al menos un síntoma indicativo de ornitosis en sus aves de compañía (plumas erizadas, disminución de la actividad, secreciones en ojos y fosas nasales, diarrea con excremento amarillo o verde y dificultad para respirar).

Se encontró una seropositividad para *C. psittaci* del 31,5 % en los trabajadores expuestos a aves incluidos en el estudio (n=17), en los que se detectaron anticuerpos tipo IgG, IgM o ambos (cuadro 2). De los siete individuos que presentaron anticuerpos IgG, cuatro tuvieron títulos positivos hasta 1:64, y tres, de 1:128. Los 14 trabajadores en los que se detectó IgM tuvieron títulos positivos de dilución 1:2.

**Cuadro 2:** Seropositividad de *Chlamydia psittaci* en los trabajadores estudiados

Seropositividad	n	%
Detección de anticuerpos contra *Chlamydia psittaci*	17	31,48
Tipo de anticuerpo detectado	n	%
IgM	10	18,5
IgG	3	5,6
IgM e IgG	4	7,4
Negativo	37	68,5
Total	54	100,0

Se exploró la asociación de la presencia de anticuerpos contra *C. psittaci* y las características sociodemográficas y de exposición a las aves en los trabajadores estudiados (cuadros 3 y 4). En el análisis bivariado se detectó una posible asociación entre la seropositividad para *C. psittaci* y ser médico veterinario (p=0,016), tener función de sacrificio y faenado de las aves (p=0,002), y tener un contacto 2 a 3 veces al mes con las aves en el entorno laboral (p=0,004) (cuadro 3). Aunque no tuvo significancia estadística, se observó una mayor proporción de individuos que presentaban anticuerpos contra *C. psittaci* y no tenían aves de compañía, en comparación con aquellos que fueron seropositivos y sí tenían aves de compañía (15 versus 2; p=0,051) (cuadro 3).

**Cuadro 3:** Análisis bivariado de la seropositividad de *Chlamydia psittaci*, y características demográficas y de exposición laboral en los trabajadores estudiados.

**Prevalencia de anticuerpos totales contra *Chlamydia psittac*** ^a^							
Variables cualitativas		No			Sí	p	
		n	%	n	%	*x* ^2^	
Ocupación	Médico veterinario	6	42,9	8	57,1	0,016	
	Zootecnista	2	50,0	2	50,0	0,407	
	Policía ambiental y ecológico	7	70,0	3	30,0	0,911	
	Trabajador en comercio de aves	17	77,3	5	22,7	0,251	
	Otras ocupaciones	5	83,3	1	16,7	0,407	
Funciones^b^	Atención médica o diagnóstica de aves	8	57,1	6	42,9	0,287	
	Limpieza de los desechos de aves	22	71,0	9	29,0	0,653	
	Transporte de aves	17	68,0	8	32,0	0,939	
	Liberación de aves	5	55,6	4	44,4	0,359	
	Comercialización de aves	16	76,2	5	23,8	0,333	
	Vigilancia y cuidado de aves	22	71,0	9	29,0	0,653	
	Sacrificio y faenado de aves	8	42,1	11	57,9	0,002	
Frecuencia de contacto laboral con las aves	Diario	22	75,9	7	24,1	0,211	
	4 a 6 días a la semana	3	75,0	1	25,0	0,772	
	2 a 3 días a la semana	8	72,7	3	27,3	0,736	
	2 a 3 veces al mes	1	16,7	5	83,3	0,004	
	1 vez al mes	2	66,7	1	33,3	0,943	
	Esporádicamente	1	100	0	0	0,494	
Síntomas de los trabajadores^c^	Presentes	23	69,7	10	30,3	0,815	
	Ausentes	14	66,6	7	33,3		
Reporte de síntomas evidenciados en	Presentes	23	65,7	12	34,3	0,547	
las aves de su lugar de trabajo^d^	Ausentes	14	73,7	5	26,3		
Ha tenido aves de compañía	Sí	14	87,5	2	12,5	0,051	
	No	23	60,5	15	39,5		
Reporte de síntomas evidenciados en las aves de compañía^e^	Presentes	4	100,0	0	0	0,383	
	Ausentes	10	83,3	2	16,7		
**Prevalencia de anticuerpos totales contra *Chlamydia psittaci*** ^*a*^							
Variables cualitativas	No			Sí			p
	n	%	n	%	n	%	*x* ^2^
Variables cuantitativas	X ± DE	Mediana (RIQ)	Mín- Máx	X ± DE	Mediana (RIQ)	Mín- Máx	P U de Mann-Whitney
Edad	39±14	34 (31-49)	18-73	36±12	32 (29-44)	23-68	0,309

DE: desviación estándar; RIQ: rango intercuartílico

^a^ Anticuerpos IgM, IgG o ambos

^b^ Un individuo puede realizar una o más funciones.

^c^ Síntomas que presentó el trabajador durante las dos últimas semanas previas al estudio.

^d^ Síntomas que el trabajador reportó observar en las aves con las que estuvo en contacto laboral durante las dos últimas semanas previas al estudio.

^e^ Síntomas que el trabajador reportó observar en las aves de compañía que tiene, durante las dos últimas semanas previas al estudio

**Cuadro 4:** Factores asociados con la seropositividad de *Chlamydia psittaci*

Variable	OR			OR ^a^		
	OR	IC_95%_	p	OR	IC_95%_	p
Médico veterinario	4,593	1,261-16,730	0,021	-	-	-
Contacto con las aves de trabajo 2 a 3 veces al mes	15,00	1,590-141,490	0,018	30,310	0,298-3.078,927	0,148
Función laboral de sacrificio y faenado de aves	5,18	1,490-17,950	0,010	**50,272**	**1,304-1.937,374**	**0,035**
Tiene contacto laboral con periquitos.	0,459	0,135-1,561	0,212	0,612	0,021-6,664	0,503
Tiene contacto laboral con patos.	0,464	0,142-1,519	0,204	0,612	0,040-9,330	0,724
Tiene contacto laboral con gallinas.	2,841	0,692-11,669	0,148	1,353	0,131-13,981	0,800
Tiene contacto laboral con bengalíes.	4,800	0,404-57,025	0,214	46,939	0,427-5.155,759	0,108
Tiene contacto laboral diario con aves.	0,477	0,148-1,534	0,214	1,001	0,063-15,945	1,000
El trabajador tiene expectoración.	2,667	0,654-10,879	0,172	0,085	0,003-2,265	0,141
Es trabajador de comercio de aves.	0,490	0,144-1,673	0,255	0,244	0,012-5,065	0,362
Tiene ave de compañía.	0,219	0,043-1,105	0,066	-	-	-
Limpieza y cuidado de aves de compañía	0,236	0,046-1,198	0,081	0,369	0,027-5,086	0,456

OR: razón de probabilidad; IC: intervalo de confianza

^a^ Variable de selección: otras profesiones u ocupaciones diferentes a las de médico veterinario (regla=0)

El análisis multivariado indicó que realizar la función de sacrificio y faenado de las aves sin ser médico veterinario es un factor de riesgo asociado con la presencia de anticuerpos contra *C. psittaci* en los trabajadores expuestos a aves (razón de probabilidad = 50; IC_95%_ = 1,3041937,374; p=0,035). Estos hallazgos demuestran la potencial exposición a *C. psittaci* en el entorno laboral de la población estudiada (cuadro 4).

En la revisión bibliográfica, solo se encontraron cuatro estudios sobre *C. psittaci* en Colombia (cuadro 5) ^25^^,^^27^^-^^29^.

**Cuadro 5:** Análisis comparativo de los estudios sobre *Chlamydia psittaci* realizados en Colombia y prevalencias detectadas

	Año de publicación	Municipio de muestreo	Detección en aves		Detección en humanos		Técnica de detección	Referencia bibliográfica
			(n)	( %)	(n)	( %)		
Monsalve, S.	2011	Manizales	36	90	4	80	ELISA indirecta	^25^
		Barranquilla	14	87	9	90		
		Montería	28	85	9	100		
		Cali	21	84	5	45		
		Victoria	19	79	3	75		
		Total en el estudio^a^	118	85	30	78		
Rivera-Osorio, S.	2018	Pereira^b^	1	-^b^	NA	NA	PCR convencional	^27^
Ocampo, M. C.	2019	Medellín	38	25,4	NA	NA	ELISA y PCR	^28^
Ruiz-Laiton, A.	2021	Bogotá	144	81,3	NA	NA	PCR convencional	^29^
Presente estudio	2023	Medellín	NA	NA	17	31,5	MIF^c^	Presente estudio

NA: no aplica

^a^ El n y el porcentaje correspondientes al total de individuos en los que se detectaron anticuerpos en todo el estudio.

^b^ En el estudio informan el muestreo de 71 aves, pero sólo se reporta el resultado de un individuo positivo.

^c^ Microinmunofluorescencia indirecta

**Figura 1:**
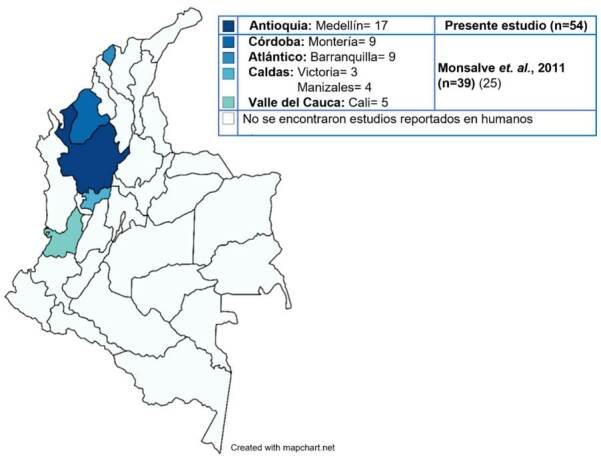
Distribución geográfica de los estudios en los que se han detectado anticuerpos contra *Chlamydia psittaci* en humanos de Colombia. El mapa de calor indica los departamentos de Colombia en los que se han detectado personas con anticuerpos para *Chlamydia psittaci*. Se indican las ciudades de muestreo, el número de personas seropositivas y el estudio en el que fueron reportadas.

Únicamente el estudio de Monsalve *et al*. (2011) muestreó humanos y encontró una seroprevalencia del 78 % en 39 trabajadores de zoológicos, expuestos a aves, provenientes de los departamentos de Córdoba, Atlántico, Caldas y Valle del Cauca (cuadro 5; figura 1). En los cuatro estudios encontrados, se reportó la presencia directa o indirecta de la bacteria en aves. En dos de ellos se encontraron prevalencias entre el 79 y el 90 % ^25^^,^^29^; en uno se indicó una prevalencia del 25,4 % ^28^ y en el último solo se detectó un ave positiva de las seis estudiadas ^27^ (cuadro 5).

El presente estudio constituye la primera evidencia de la circulación de *C. psittaci* en trabajadores expuestos a aves en el departamento de Antioquia y el segundo reporte en el país (figura 1).

## Discusión

En esta investigación, se encontró una seropositividad del 31,5 % de anticuerpos contra *C. psittaci* en individuos expuestos laboralmente a aves en Medellín. El único estudio previo encontrado sobre *C. psittaci* en humanos en el país ^25^ encontró una prevalencia superior a la hallada en el presente estudio (78 %). La discrepancia entre estos resultados podría deberse a una posible sobreestimación de los niveles de anticuerpos anti-*C. psittaci* en el estudio de Monsalve *et al*., dada la técnica inmunológica que se usó para su detección (ELISA). A diferencia de la microinmunofluorescencia (usada en el presente estudio) ^26^, ELISA emplea como antígeno a la proteína rMOMP (*Major Outer Membrane Protein*). Esta proteína posee epítopos conservados entre especies de la familia *Chlamydiaceae* y puede generar posibles reacciones cruzadas interespecies ^30^.

Estudios de otros países también han demostrado la exposición a *C. psittaci* de personas en contacto con aves en su entorno laboral. En el estudio de Lugert *et al*., realizado en Alemania y publicado en 2017, se determinó la seroprevalencia de *C. psittaci* en 231 trabajadores de granjas de patos durante tres años distintos, la cual varió entre el 7,1 y el 8,3 % ^31^.

En el 2019, se reportó un brote de psitacosis en trabajadores de plantas de sacrificio de aves de corral de Estados Unidos, donde 29 trabajadores fueron hospitalizados, tres de los cuales tuvieron que ser hospitalizados en unidades de cuidados intensivos ^32^. En ese mismo año se publicó un estudio en Argentina en el que, luego de un seguimiento de dos años a trabajadores de reservas naturales de Buenos Aires, se encontró una prevalencia directa e indirecta de *C. psittaci* del 28,6 % (Favier P, Arias S, Lara C, Wiemeyer G, Crivelli A, Ludvik H, *et al*. *Chlamydia psittaci* en trabajadores de reservas naturales de la ciudad de Buenos Aires: transmisión ante la aparente normalidad. Seguimiento a dos años. En: XIX Congreso SADI - 2019. Sociedad Argentina de Infectología; 2019).

Aunque la mayoría de los resultados reportan la circulación de *C. psittaci* en aves y personas en contacto con éstas, también se ha evidenciado la presencia de la bacteria en otros animales, como hurones ^5^, y en trabajadores de granjas expuestos a ganado vacuno y porcino ^9^.

En este estudio se encontró que el ejercer funciones de sacrificio y faenado de las aves sin ser médico veterinario, es un factor de riesgo para el desarrollo de anticuerpos contra *C. psittaci*. Aunque el intervalo de confianza del 95 % calculado para la razón de probabilidad de dicho factor, muestra un amplio rango de imprecisión, este puede explicarse por el reducido tamaño de la muestra. No obstante, el resultado es confiable y las variables de confusión se controlaron (material suplementario).

Este hallazgo sugiere que los trabajadores estudiados pudieron tener una mayor exposición a *C. psittaci* por medio de las labores de sacrificio y faenado, lo cual coincide con estudios previos, en los cuales se ha identificado una mayor frecuencia de psitacosis en trabajadores que ejercen esas funciones ^32^^,^^33^. Según los CDC, quienes cumplen labores de sacrificio y faenado son las personas que están en mayor riesgo de contraer la infección, debido a que, en algunos pasos del proceso, hay una importante exposición a las formas infecciosas de *C. psittaci*^34^.

La mayoría de la población estudiada eran personas con formación académica o laboral diferente a las del área de la salud y, como se expresó anteriormente, el sacrificio y faenado representó un factor de riesgo ligado a la condición de no ser médico veterinario. Lo anterior puede deberse a que las profesiones u ocupaciones diferentes a las del sector salud tienen una capacitación más reducida frente al riesgo biológico, lo que podría llevar a prácticas con mayor exposición en los trabajadores. Se ha demostrado que el desconocimiento de la infección y sus formas de transmisión para adoptar conductas preventivas en el ambiente laboral aumenta la probabilidad de adquirir la psitacosis ^35^.

La presencia de anticuerpos contra *C. psittaci* en la población estudiada no se asoció con factores sociodemográficos; tampoco se encontró un patrón o asociación con los tipos de aves a los que se estuvo expuesto laboral o domésticamente. Sin embargo, el hecho de que la mayoría de los sujetos seropositivos no tenía aves de compañía, que su contacto con ellas se producía en el entorno laboral, además de la clara asociación de la seropositividad con labores de sacrificio y faenado, son factores que indican que la exposición de la población evaluada pudo ser ocupacional y recalca la importancia de reforzar las capacitaciones en manejo adecuado del riesgo biológico en personas con exposición laboral a las aves ^19^^,^^34^^,^^36^.

La revisión bibliográfica encontró solo cinco reportes de *C. psittaci* en Colombia. Estos hallazgos muestran el gran desconocimiento que hay sobre *C. psittaci*, sobre la infección que causa en animales y en humanos, y sobre sus implicaciones en la salud pública del país. De los estudios colombianos que han analizado en las aves la presencia directa o el contacto con *C. psittaci*, dos informaron prevalencias entre el 79 y el 90 % ^25^^,^^29^, mientras que el estudio de Ocampo *et al*. reportó una prevalencia del 25,4 % ^28^. La diferencia en estas prevalencias puede deberse a que, en el último, se analizaron muestras de aves silvestres, mientras que, en los otros dos estudios, se hicieron muestreos de aves en cautiverio.

Esta diferencia también fue expuesta en un estudio en Taiwán en el 2019 ^37^. Las elevadas prevalencias halladas en las aves de diferentes centros del país, evidencian el gran potencial de riesgo de exposición laboral a la bacteria por el contacto estrecho con aves ^37^^,^^38^. Esto se refuerza con las seropositividades halladas en los trabajadores expuestos analizados en este estudio y en el de Monsalve *et al*., que son superiores a las de reportes internacionales como el de Favier *et al*. (Favier P, Arias S, Lara C, Wiemeyer G, Crivelli A, Ludvik H, *et al*. *Chlamydia psittaci* en trabajadores de reservas naturales de la ciudad de Buenos Aires: transmisión ante la aparente normalidad. Seguimiento a dos años. En: XIX Congreso SADI - 2019. Sociedad Argentina de Infectología; 2019) ^31^.

Esta investigación tiene limitaciones. El diseño descriptivo transversal y el reducido tamaño de la muestra no permiten hacer inferencias explícitas de causalidad o relación, por lo que los resultados se deben valorar como una exploración de la posible asociación de las variables analizadas y la presencia de anticuerpos contra *C. psittaci*.

Por otra parte, en el estudio no se indagó por prácticas de prevención de la infección en los trabajadores, por ejemplo, el uso de equipos de protección personal. Tampoco se investigó sobre su exposición a otros animales diferentes a las aves que también pueden ser reservorios de la bacteria. Por último, no se hizo un muestreo de las aves de compañía y del entorno laboral con el que estaban en contacto los sujetos estudiados. Estas situaciones generan una limitación importante en la evaluación de la exposición de los trabajadores incluidos en el estudio.

El presente estudio constituye la primera evidencia de la circulación de *C. psittaci* en trabajadores expuestos a aves en el departamento de Antioquia y el segundo reporte en el país. Tomando en conjunto los resultados de esta investigación, se sugiere realizar vigilancia epidemiológica del personal que trabaja con aves en la ciudad y el país, bajo la estrategia de “una sola salud” (*One Health*); asimismo, intensificar la capacitación sobre riesgos biológicos y métodos de prevención y protección personal en los trabajadores expuestos ^20^. Además, se recomienda que el personal de la salud considere la posibilidad de infección causada por *C. psittaci* ante la consulta por enfermedades febriles respiratorias en trabajadores en contacto con aves.

## Figures and Tables

**Figura suplementaria:**
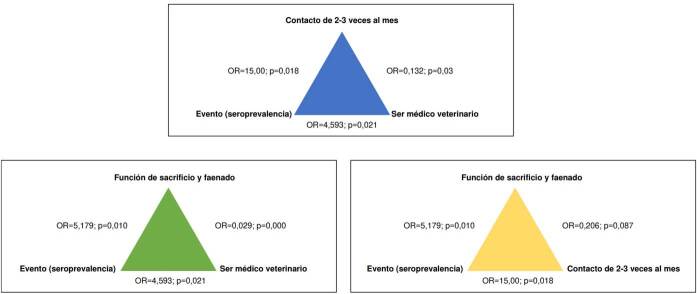
1. Análisis de confusión
